# Does a bilateral polypropylene mesh alter the duct deferens morphology, testicular size and testosterone levels? Experimental study in rats^1^

**DOI:** 10.1590/s0102-865020200020000001

**Published:** 2020-04-17

**Authors:** Sérgio Henrique Bastos Damous, Luciana Lamarão Damous, Jocielle dos Santos Miranda, Edna Frasson de Souza Montero, Cláudio Birolini, Edivaldo Massazo Utiyama

**Affiliations:** I MD, PhD, Division of General Surgery and Trauma, Department of Surgery, Hospital das Clínicas da Faculdade de Medicina da Universidade de São Paulo (HC/FMUSP), Brazil. Conception and design of the study, technical procedures, analysis of data, manuscript writing.; II MD, PhD, Department of Obstetrics and Gynecology, Faculdade de Medicina da Universidade de São Paulo (FMUSP), Brazil. Analysis and interpretation of data, histological images, manuscript writing.; III PhD, Division of General Surgery and Trauma, Department of Surgery, HC/FMUSP, Sao Paulo-SP, Brazil. Conception and design of the study, technical procedures, analysis of data.; IV Associate Professor, Surgical Gastroenterology Division, Department of Surgery, UNIFESP, Sao Paulo-SP, Brazil. Conception of the study, critical revision.; V MD, PhD, Division of General Surgery and Trauma, Department of Surgery, HC/FMUSP, Sao Paulo-SP, Brazil. Critical revision.; VI Full Professor, Division of General Surgery and Trauma, Department of Surgery, HC/FMUSP, Sao Paulo-SP, Brazil. Conception and design of the study, critical revision, final approval.

**Keywords:** Hernia, Surgical Mesh, Polypropylenes, Vas Deferens, Testis, Rats

## Abstract

**Purpose:**

To evaluate the effect of a PP mesh on duct deferens morphology, testicular size and testosterone levels.

**Methods:**

Forty adult male rats were distributed into groups: 1) no surgery; 2) inguinotomy; 3) mesh placed on the duct deferens; and 4) mesh placed on the spermatic funiculus. After 90 postoperative days, the inguinal region was resected, and blood samples were collected for the measurement of serum testosterone (pg/dl). The ducts deferens were sectioned in three axial sections according to the relationship with the mesh — cranial, medial and caudal. The wall thickness and duct deferens lumen area were measured.

**Results:**

The morphology of the duct deferens was preserved in all groups. The mesh placement did not alter this morphology in any of the analyzed segments. Surgery, with or without mesh placement, did not alter the morphology, wall thickness or lumen area (p>0.05). In all operated groups, serum testosterone levels were similar (p>0.05) but there was a decrease in testicle size (p<0.05).

**Conclusion:**

Surgery, with or without mesh placement, did not alter the morphology of the duct deferens and, although this treatment resulted in testicular size reduction, it did not affect serum testosterone levels.

## Introduction

The implantation of a synthetic mesh for the correction of inguinal hernias created a new paradigm in the treatment of this condition, and its use today is a consensus in the world literature. The type of mesh most commonly employed is polypropylene (PP), and its use causes intense fibroplasia and reinforcement of the abdominal wall in the inguinal region, with consequent reduction in the rates of relapse to less than 1%, in addition to a reduction in the cumulative risk mainly from the first year, compared to the technique without a mesh^1-3^.

On the other hand, the use of meshes can cause complications due to the intense inflammatory response that is induced, such as inflammation around the screen, chronic pain in up to 15-30% of patients^5^, and impairment of male fertility^6-8^.

In experimental models, it was observed that the use of PP meshes causes morphological changes and even obstruction of the vas deferens^9^. Other changes described are decreased testicular perfusion and decreased spermatogenesis^10^. In humans, there are several case reports and clinical studies with divergent results regarding fertility preservation^6-8,11^, while doubts remain about the real impact of mesh use on male fertility, mainly in bilateral corrections.

Currently, the most common surgical techniques used for inguinal hernioplasty are the Lichtenstein and laparoscopic techniques. In the first method, the mesh surrounds the spermatic funiculus at the level of the deep inguinal ring, while in the second, the mesh is in direct contact with the duct deferens and gonadal vessels after their parietalization. Therefore, this study aimed to evaluate the deferent duct and the testicle of rats after the implantation of a PP synthetic mesh, with a comparison of the two surgical techniques most used for inguinal hernioplasty.

## Methods

This study was carried out in the Medical Research Laboratory (LIM-62) of the Division of Clinical Surgery III (DCC III) of Hospital das Clínicas da Faculdade de Medicina da Universidade de São Paulo (HC/FMUSP) after approval by the Ethics Committee for Use of Animals in Research of FMUSP under number 089/15. All animals were handled according to the principles of the National Institute of Health (1985) and The American Physiological Society (1995) for the care, handling and use of laboratory animals.

A total of 40 male adult Wistar rats weighing between 300 g and 400 g were used from the FMUSP laboratory. The animals were maintained in LIM-62 throughout the experiment, they were kept in individual ventilated cages with a controlled temperature and a light-dark period without restriction of water and feed intake.

**Figure 1:**
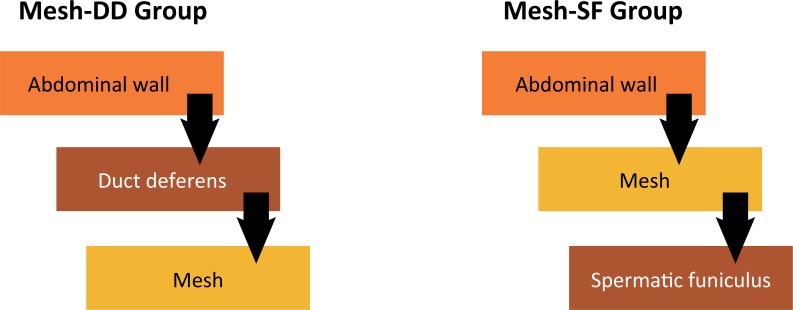
Illustrative diagram of the location of the mesh, showing its relationship with the abdominal wall and the structures of the spermatic funiculus.

The animals were distributed into four study groups with 10 animals each, as follows: 1) No surgery (No): intact animals; 2) Inguinotomy (I): animals submitted to a bilateral 3 cm incision, spermatic funicular exposure and dissection of the vas deferens without mesh implant and posterior wall closure; 3) Mesh placed on the duct deferens (Mesh-DD), so that the layout of the mesh simulates the laparoscopic technique; and 4) Mesh placed on the spermatic funiculus (Mesh-SF), in which the mesh layout simulated the Lichtenstein technique (Fig. 1). In all operated animals, the surgery was bilateral.

### Surgical procedure

The animals underwent general anesthesia with intraperitoneal injection of ketamine (50 mg/kg) combined with xylazine (10 mg/kg) in the same syringe and, immediately after the anesthetic effect, abdominal and inguinal bilateral trichotomy with local antisepsis were performed. Bilateral inguinotomy was performed by means of a 3 cm extension incision. In the mesh groups, a fragment of a high molecular weight PP mesh with small pores measuring 2 cm wide by 2 cm in length was used.

In the Mesh-DD group, the spermatic funiculus was dissected, and the duct deferens was isolated from the other structures. The mesh was placed in direct contact on the duct deferens and fixed with four separate points with a polyglycolic acid 4.0 thread, leaving the duct deferens between the mesh and the abdominal wall, simulating the parietalization of the elements of the spermatic funiculus.

In the mesh-SF group, the mesh was placed under the spermatic funiculus at the level of the deep inguinal ring and fixed with four separate points on the abdominal wall with a polyglycolic acid 4.0 thread, leaving the funiculus above the mesh and the abdominal wall. A 0.5 cm-wide slit was made on the mesh in the cranial part to surround the spermatic funiculus, simulating the Lichtenstein technique.

In all operated groups, the skin was sutured with separate stitches with 4.0 monylon thread.

### Collection of surgical specimens and histological preparation

After 90 days, all animals were anesthetized again by the same technique described above for resection of surgical specimens and blood sample collection. The analysis of the duct deferens and measurement of the testicular size was standardized on the right side in all animals. Blood samples were collected from the venous plexus of the spermatic cord for serum testosterone dosage in duplicate (Testosterone ELISA Kit Abcam108666, USA). After sample collection, the animals were euthanized by a lethal dose of the anesthetics used.

The testicle was measured with a pachymeter in the longitudinal and transverse directions to calculate its size. Then, the right inguinal region was dissected in a block, and the duct deferens were isolated and sectioned in three axial segments, according to the relation with the mesh: 1) cranial: 1 cm above the mesh; 2) medial: in contact with the mesh; 3) caudal: 1 cm below the mesh. After the sample collection, the animals were submitted to euthanasia by means of a lethal dose of the anesthetic used.

The duct deferens segments were immediately fixed in 4% paraformaldehyde for at least 24 h. Following fixation, the samples were dehydrated, paraffin-embedded, serially sectioned at 5 μm, and mounted on glass microscope slides. Routine hematoxylin and eosin (HE) staining was performed for histological examination with light microscopy.

### Morphological and morphometric analyses

All images of the sections were obtained using an image acquisition software system (Leica DM2500; LEICA, Wetzlar, Germany). Images of the sections were obtained using an image acquisition software system (Leica DM2500), and measurements were made using Leica QWin V3 software. Two independent investigators blind to the experimental treatments performed all of the analyses under a microscope (LEICA).

After capturing the images at x50 magnification, morphological evaluation was achieved through descriptive analyses of the duct deferens. For morphometric analysis, measurements of wall thickness (four measurements per slide) and measurement of the area of light of the vas deferens were performed.

### Statistical analysis

The results are expressed as the mean±standard deviation of the mean. Two-way ANOVA was utilized to analyze the measurements of the duct deferens and one-way ANOVA to analyze the testicular size and testosterone levels. All statistical analyses were performed using GraphPad Prism 8.0 (GraphPad Software, Inc., CA, USA); p values lower than 0.05 were considered significant.

## Results

### Morphology

**Figure 2:**
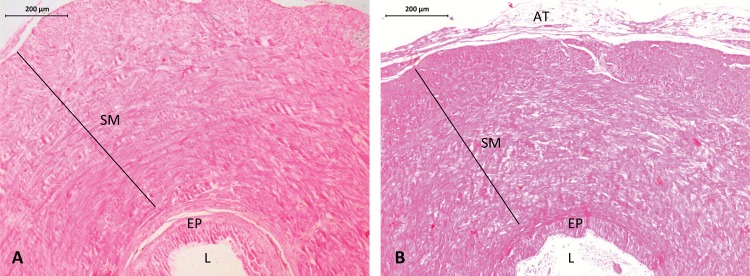
Photomicrographs of a representative rat duct deferens with HE staining – axial section of the segment in contact with the mesh in animals that received inguinotomy without mesh placement (A) and animals that underwent mesh placement on the duct deferens (B). Mesh placement did not induce any changes in morphology. SM=Smooth musculature. EP=Epithelium. L=Lumen. AT=Adipose tissue (x100).

In groups No and I, all segments of the duct deferens were preserved in morphology. The duct deferens, from the lumen, consisted of pseudostratified epithelium and stereocilia, surrounded by a thin layer of connective tissue and a thick layer of smooth musculature, externally surrounded by adipose tissue and connective tissue. In the mesh groups (Mesh-DD and Mesh-SF), there was no difference in the morphology in the segments in contact with the mesh with the cranial and caudal segments (Fig. 2).

### Duct deferens morphometry

#### Wall thickness

**Figure 3:**
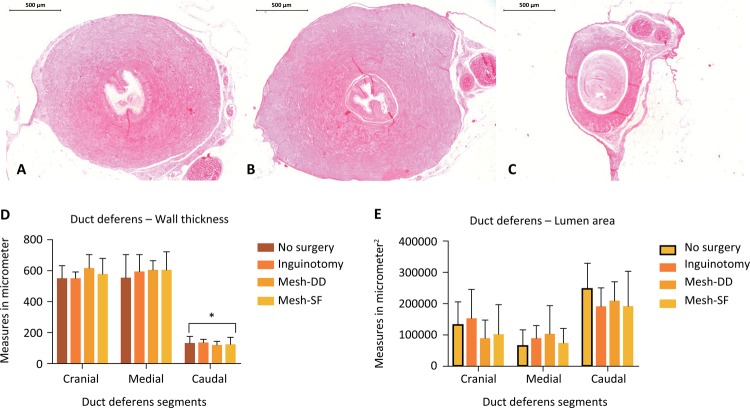
Photomicrographs of a representative rat duct deferens with HE staining – axial section of segments in according to the relation with the mesh: (A) cranial: 1 cm above the mesh; (B) medial: in contact with the mesh; and (C) caudal: 1 cm below the mesh. (D) In all animals there was an anatomical reduction in the wall thickness in the caudal segment (*p<0.05 caudal *vs*. medial and cranial). (E) There was a tendency of increasing the lumen area showed in the caudal segment in all groups, but there was no significant difference (p>0.05). Surgery, with or without mesh placement (I, Mesh-DD and Mesh-SF), did not alter the duct deferens anatomy.

In nonoperated animals (No), there was an anatomical reduction in the wall thickness of the duct deferens in the caudal segment (p<0.05) caudal vs. medial and cranial). Surgery, with or without mesh placement (I, Mesh-DD and Mesh-SF), did not alter this duct deferens anatomy (Table 1, Fig. 3).

**Table 1:** Measurement of the wall thickness of the cranial (1 cm above the mesh), medial (in contact with the mesh) and distal (1 cm below the mesh) segments of the duct deferens of rats after 90 days of PP mesh implantation in the inguinal region.

Wall thickness	No Surgery	Inguinotomy	Mesh-DD	Mesh-SF
Cranial	551.1±79.7	557.5±35.7	618.8±85.3	575.5±100.4
Medial	557.5±146.5	593.5±112.2	605.4±57.6	604.4±117.4
Caudal^*^	135±37.7	138.2±18.2	122.5±21.5	125.5±45.9

^*^p<0.05. Two-way ANOVA.

#### Lumen area

The lumen area of the duct deferens showed a tendency to increase in the caudal segment in all groups, but there was no significant difference (p> 0.05). Among the operated animals, there was no difference in lumen area, regardless of the use of the screen (p>0.05) (Table 2, Fig. 3).

**Table 2:** Measurement of the lumen area of the cranial (1 cm above the mesh), medial (in contact with the mesh) and distal (1 cm below the mesh) segments of the duct deferens of rats after 90 days of PP mesh implantation in the inguinal region.

Lumen area	No Surgery	Inguinotomy	Mesh-DD	Mesh-SF
Cranial	133.819	153.399	91.104	103.467
Medial	67.852	90.961	102.348	73.354
Caudal	249.571	193.461	208.743	191.788

p>0.05. Two-way ANOVA.

## Testicular analysis and hormonal dosage

**Figure 4:**
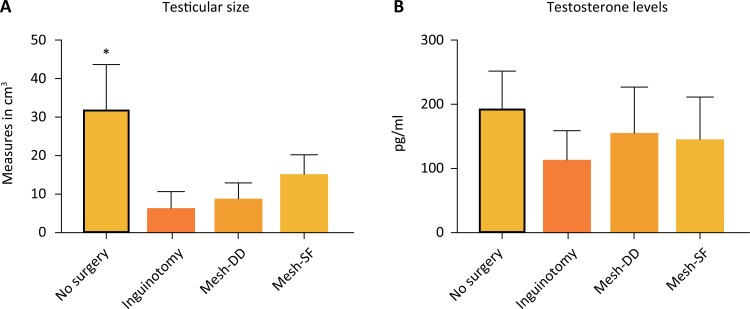
Measurement of testicular size (A) and testosterone serum levels (B). Surgery, with or without the use of the mesh, promoted a reduction in testicular size; however, serum testosterone levels were similar among all groups. *p<0.05 No *vs*. I; Mesh-DD; Mesh-SF.

Surgery, with or without the use of the mesh, promoted a reduction in testicular size (p<0.05 No vs. I; Mesh-DD; Mesh-SF); however, serum testosterone levels were similar among all groups (p>0.05) (Table 3, Fig. 4).

**Table 3:** Testicular volume and testosterone serum levels in rats after 90 days of PP mesh implantation in the inguinal region.

	No Surgery	Inguinotomy	Mesh-DD	Mesh-SF
Testicular size^#^	32.2±11.5^*^	6.5±4.2	9±3.9	15.2±5
Testosterone^W^	194.6±58	116.5±44	157.9±70.5	148.5±63

^#^Medida em cm^3^; ^W^Medida em ng/ml

*p<0.05

## Discussion

Hernia repairs with or without mesh are both proven effective in the treatment of inguinal hernias. On one hand, compared to nonmesh repairs, mesh repairs reduce the rate of hernia recurrence and involve a shorter operation time and faster return to normal activities. On the other hand, testicular complications probably occur slightly more often in mesh repairs and show nearly equivocal results^12^.

The PP mesh is used both by inguinotomy and laparoscopically and causes a foreign-body-type inflammatory reaction around it, followed by fibrosis and scar formation in the preperitoneal space, which can cause discomfort and chronic pain. More recently, use of the mesh has been related to infertility, a rare and underestimated iatrogenic complication. However, the causal relationship with vas deferens and/or testicular changes still needs to be proven^13^.

A recent systematic review with 7 randomized clinical trials suggests that hernia repair with mesh either in an open or a laparoscopic procedure has no significant effect on male fertility^14^. It seems that mesh hernia repair causes only transitory changes in testicular blood flow and no clinically significant autoimmune reactions^15^. Whether changes in flow parameters remain in the late postoperative period and whether they have an impact on complications should be evaluated in further clinical and experimental studies^16^.

There are currently few experimental studies analyzing the impact of herniorrhaphy on male fertility. Most of the studies found in the literature use different surgical techniques on each side of the inguinal region in the same animal or evaluate different types of meshes on each side, in addition to analyzing only the region of the duct deferens in contact with the mesh. In this experimental model, we used the same type of PP mesh in all animals, and we performed the same surgical technique in both inguinal regions of the same animal, simulating the two techniques most used in clinical practice. The analyses were performed in different segments of the duct deferens: cranial (above the mesh), medial (on the topography of the mesh) and caudal (below the mesh). Thus, it was possible to observe in all groups that in the caudal segment of the duct deferens, there is a reduction in wall thickness and increased light, which shows that surgery, with or without a mesh, did not alter the histomorphometry of the different segments of the duct deferens. These findings show the innocuous effect of the mesh in the analysis of this variable. Therefore, we believe that the results of experimental models that evaluate the effects of the mesh without having a control group for comparison can be questioned, even if the purpose of these studies is to compare different types of meshes with each other. Once it is proven that there is no functionally relevant change in the duct deferens either upstream (above) or downstream (below) of the segment with the mesh, further studies can be performed only in the segment in contact with the mesh to optimize the analyses.

There are reports in the literature regarding alterations of the duct deferens with the use of the mesh, which differs from our findings. These authors observed that there was a reduction in the wall thickness in the caudal segments and in contact with the mesh, being more pronounced in the first segment, which was dilated, with an increase in the lumen area and sperm accumulation. However, these conditions do not seem to alter testicular function since no alteration was observed in the testicles of these animals^17^.

Surgery, with or without mesh implantation, promoted a reduction in testicular volume. Although there was no significant difference between the operated groups, the use of the mesh alone was not an aggravating factor, since the largest reduction occurred in the group without the mesh (I), which presented a volume of approximately 20% of the volume of the control group. The mesh groups, in turn, presented volumes that were 53% and 71% of the volume of the control group (Mesh-DD and Mesh-SF, respectively).

Theoretically, the greatest inflammatory reaction is expected in the tissue of mesh groups, but in the duct deferens, no changes were observed, and in the testicle, volume changes were observed in all the operated groups. This finding makes us suppose that not only mechanical factors, but also humoral and inflammatory factors related to the local surgical trauma, are involved in this process, and therefore, different alterations in each organ are possible (maintenance of the duct deferens and testicular atrophy) that do not necessarily compromise functionality. This fact can be corroborated by the similar serum testosterone levels in all groups, regardless of the testicular volume reduction.

Initial studies in this line of research have already described histological changes in a certain segment of the duct deferens without histological or functional repercussion in the testicles^17^. Vasography exams performed 90 days after PP mesh implantation confirmed the patency of the duct deferens of rats, with no change in the lumen of the duct deferens, testicle weight or serum testosterone levels. However, a limiting factor of this experimental model is that the contralateral side was used as a control^18^. After 180 days, vasography revealed relevant obstructions (>75% of the lumen diameter) located at the mesh margins in half of the cases but did not impact spermatogenesis; however, in this study, there was no control group without surgery or surgery without mesh implantation^19^. In a later study involving a histological analysis of rat testicles 90 days after PP mesh implantation, the authors described an intense congestion in the necrotic tissue of the seminiferous tubules with significant reduction in spermatozoa type a and b and an increase in antisperm antibodies in serum^20^. These data show that, in a more sensitive analysis, there may be changes in spermatogenesis that do not necessarily negatively impact fertility.

Other experimental models have been described in the literature comparing the effect of different types of meshes (including PP) on the fertility of young and adult rats in the long term (90 to 180 days postoperatively) with divergent results. The majority of these studies do not show a negative association even with the PP mesh^11,21^, which promotes increased activation of macrophages and, consequently, a more intense inflammatory reaction than low molecular weight screens^23^. However, all these models fail to include a group without mesh implantation for comparison^18-22^.

Changes in testicular tissue metabolism have been described in rats with PP mesh implantation, such as an increase in testicular nitric oxide in the long term (180 days) without, however, altering LH and FSH levels or inducing apoptosis^24^. An early in vivo evaluation of the angiogenic and inflammatory host tissue response 14 days after implantation onto the striated muscle tissue of hamsters showed that the PP mesh was surrounded by newly formed granulation tissue, fully collagen fibers and that activated leukocytes were accumulated, particularly in blood vessels growing inside the mesh implants^25^. The analysis of the inflammatory response and tissue reorganization through the study of collagen fibers and tissue neovascularization around the mesh can bring more information about the adaptive mechanisms to the mesh and the new microenvironment that develops around it.

It should be noted that a limiting factor of the present study was the lack of a testicular-morphology or spermatogenesis analysis. On the other hand, the testicular volume and serum levels of testosterone were analyzed as a testicular functional evaluation. We observed that the surgical manipulation itself, and not the use of the mesh, is related to testicular volume reduction but without functional impairment, as the testosterone levels remained similar to those in the nonoperated animals.

Models in medium-sized animals such as pigs and dogs show different results than those observed in rodents. In earlier analyses in pigs - 7, 14, 21, 28, and 35 postoperative days - the PP mesh repair by the Lichtenstein technique reduced arterial perfusion, the testicular temperature and spermatogenesis but increased the testicular volume by 10%. In this study, the control group was the contralateral region of the same animal operated on by another surgical technique (Shouldice). In the rabbit model, the analysis was performed after 90 days and showed that the inflammatory changes were less evident and were directly related to the duration of the postoperative period. The authors suggest that this difference may be related to the greater protection of the structures of the spermatic cord by the cremasteric muscle, which was spared in the rabbits and not in the pigs. A relevant difference between the two models is that in the pigs, the authors sectioned the transverse fascia to create a defect similar to the hernial sac that was immediately corrected with the placement of the screen in the same surgical time^10^. In our opinion, the results described in pigs are questionable because the sample analyzed is very small, with only three animals evaluated at each time.

In dogs operated on by the same technique bilaterally, after 60 days, the PP mesh led to a more intense chronic inflammatory reaction and a significant reduction in the lumen diameter of the duct deferens. A failure of this study is the lack of homogeneity of the sample, since the authors did not report the origin of the animals and cited that they were apparently healthy. Some animals were caught on the street, not taking into account age and coexisting diseases, which could have strongly influenced the results^26^.

Although there are limitations in the experimental models, due to the characteristics inherent to each species, these investigations contribute greatly to translation to humans because such models allow us to perform analyses that are otherwise impossible for ethical reasons. Future studies should deepen the analysis of the effect of the PP mesh on the functionality of these organs and the real impact on male fertility.

## Conclusion

Surgery, with or without mesh placement, did not alter the morphology of the duct deferens, and although this procedure resulted in testicular size reduction, it did not affect serum testosterone levels. Future studies should deepen the analysis of the effect of the PP mesh on the functionality of these organs and the real impact on male fertility.

## Footnotes

1 Research performed at Division of General Surgery and Trauma, Department of Surgery, Hospital das Clínicas da Faculdade de Medicina da Universidade de São Paulo (HC/FMUSP), Brazil.
